# Vitamin B12 Deficiency due to Chlorofluorocarbon: A Case Report

**DOI:** 10.1155/2010/691563

**Published:** 2011-02-27

**Authors:** Hemlata Bhaskar, Rekha Chaudhary

**Affiliations:** ^1^Department of Medicine, The University of Toledo, 3000 Arlington Avenue Toledo, OH 43614, USA; ^2^Division of Hematology and Oncology, The University of Toledo, 3000 Arlington Avenue Toledo, OH 43614, USA

## Abstract

*Background*. Vitamin B12 is vital for optimal functioning of various organ systems but more importantly the central nervous system and the hematological system. Deficiency of vitamin B12 clinically manifests as excessive daytime fatigue, memory difficulties, encephalopathy, myelopathy, peripheral neuropathy, and optic neuropathy. In occupational medicine, vitamin B12 deficiency has been reported with exposure to nitrous oxide in health care workers. However, not much is known about exposure to Freons in other industries and vitamin B12 deficiency. *Aim*. We are reporting a case of vitamin B12 deficiency in the setting of exposure to chlorofluorocarbon (CFC) gases. *Case Report*. A 55-year-old male refrigerator mechanic experienced recurrent visual symptoms, which included diplopia and blurring. A complete workup was done and was significant of vitamin B12 deficiency. However, his B12 levels were refractory to supplementation. Appropriate precautions at workplace improved patient's symptoms and were associated with significant improvement in B12 levels. *Conclusion*. To the best of our knowledge, this is the first reported case of vitamin B12 deficiency (that remains refractory to supplementation) in the setting of exposure to Freon gases.

## 1. Introduction

Vitamin B12 is essential for optimal functioning of various organ systems including the brain, spinal cord, peripheral or cranial nerves, and blood cell production. Vitamin B12 (cyanocobalamin) deficiency produces dementia, peripheral neuropathy, subacute combined degeneration of the spinal cord, nutritional amblyopia (visual loss), and cognitive dysfunction [1]. Neurologic abnormalities may precede the development of macrocytic anemia. B12 deficiency has been reported in health care workers exposed to nitrous oxide. However the prevalence of cyanocobalamin deficiency remains unknown in other industries. In the current report, we discuss a case of vitamin B12 deficiency presenting with neurological symptoms, which was found to be associated with chlorofluorocarbon gases (in the refrigerator industry).

## 2. Case

A 55-year-old male presented with 2-3-month history of intermittent visual symptoms including diplopia and blurring. Each episode lasted approximately 30 minutes. Patient denied any prior history of experiencing similar visual symptoms. No other symptoms accompanied the visual disturbance. Each episode occurred at the end of long tiring day at work. He had history of hypertension, hyperlipidemia, and exercise-induced asthma. He was married and lived with his wife. He denied smoking and drank alcohol occasionally. His medications included lisinopril and hydrochlorothiazide, celecoxib, lovastatin, and zolpidem.

**Figure 1:**
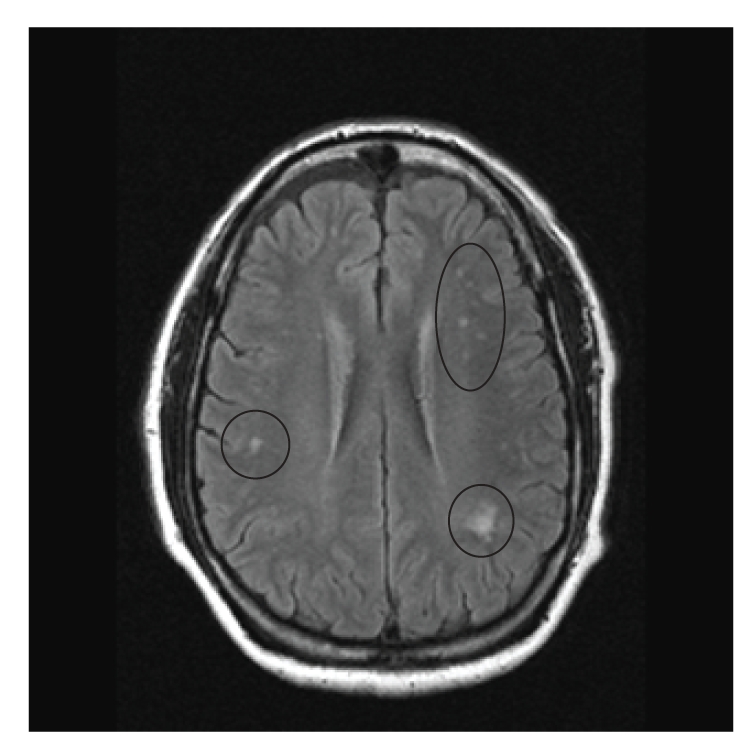
MRI image of the brain in an axial view showing the “precontrast FLAIR image”. Note the abnormal lesions (circled) in the per ventricular area suggesting white matter pathology.

**Figure 2:**
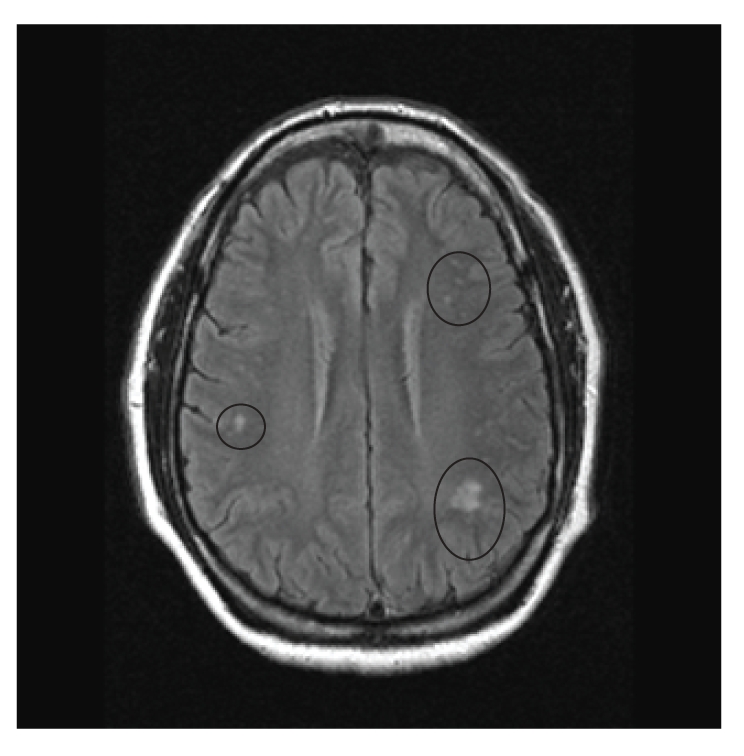
MRI image of the brain in an axial view showing the “post-contrast FLAIR image”. Note the abnormal lesions (circled) in the per ventricular area do not show any enhancement with contrast injection.

**Figure 3:**
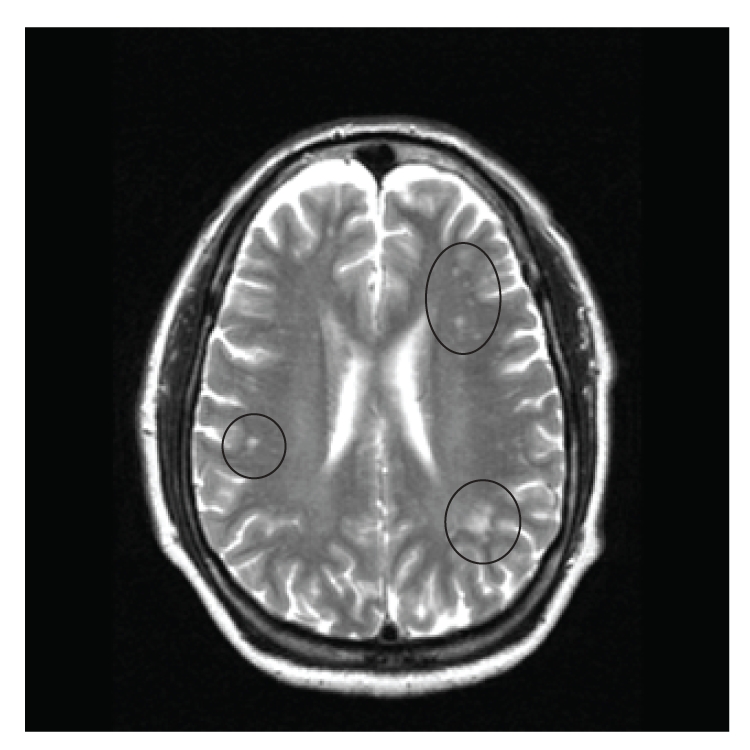
MRI image of the brain in an axial view showing the “pre-contrast T2 image”. Note the abnormal lesions (circled) in the per ventricular area suggesting white matter pathology.

He ran a business of repairing refrigerators. His father died at the age of 84, suffered from heart disease, had brain tumor and optic nerve tumor which was removed, and was left with permanent visual disturbance. His mother also died at the age of 84 and had h/o open heart surgery, breast cancer, and diabetes mellitus. His siblings had cardiac disease and diabetes mellitus. His physical exam was normal. Upon initial presentation to his primary care physician, an imaging study of the brain was requested. An MRI of brain was obtained which showed multiple areas of T2 hyper-intensity in the subcortical white matter especially in periventricular region. Figures 1 and 2 show pre- and post-contrast FLAIR images and Figure 3 shows T2 weighted image suggesting white matter pathology. Based on the neuroimaging, a demyelinating process was hypothesized. 

This prompted a consultation with neurology. At neurology clinic he denied any headache, episodes of loss of consciousness, memory loss, and gait disturbances. His physical exam was within normal limits. A specimen of peripheral blood was obtained to check vitamin B12 levels, ESR, antinuclear antibody, PTT, antimicrosomal antibody, antiphospholipid antibody, and Lyme's serology. His vitamin B12 level was 59 pg/mL. His other labs were normal. His 25 hydroxy-vitamin D level was 22 ng/mL, homo-cysteine 13.9 micro mol/L, and methylmalonic acid 0.82 micro mol/L. He was started on cyanocobalamin injections daily for one week, then once a week for one month, then once a month. Vitamin D supplements were also started. His vitamin B12 levels were checked after two months, which continued to be in range of 50–80 pg/mL. 

Hematology was consulted and there his review of systems was also positive for increased appetite, moderate fatigue, memory problems, numbness in his hands when he sleeps and muscle stiffness. He also complained of palpitations, increased urinary frequency. Due to refractory vitamin B12 levels, he was thought to have pernicious anemia, anti parietal cell antibody and anti-intrinsic factor antibody levels were checked. His antiparietal cell antibodies were positive. However, the fact that low levels of B12 persisted despite parenteral supplementation suggested that pernicious anemia was an unlikely explanation for persistent low B12 levels. Gastroenterology was consulted. An esophagogastroduodenoscopy revealed minimal gastritis. Helicobacter pylori special stain was negative. His intrinsic factor antibody was negative. 

In conjuncture with previously reported cases of B12 deficiency in health care workers due to exposure to nitrous oxide [8], possible role of CFC and other Freon gases was hypothesized especially in the light of his occupation. To determine this association, patient was recommended to wear an tightly fitting naso-oral mask when working and his B12 supplementation was continued. On follow-up visit his symptoms had resolved and significant improvement was noted in his B12 level to 554 pg/mL. To validate this hypothesized association, the patient was asked not to wear the mask while at work and significant drop in B12 levels was noted on followup.

## 3. Discussion

Chlorofluorocarbon, an organic compound that contains carbon, chlorine, and fluorine where as hydrocholoroflurocarbon, a related organic compound, in addition contains hydrogen, These compounds are commercially available under the trademark freon and are commonly used as refrigerants, propellants, and solvents. In recent times, growing interest in them has been shown as they contribute to ozone depletion [2]. Exposure to Freon gases has been associated with cardiac arrhythmias, elevated chloesterol, secondary hypertension, and renal dysfunction [3, 4]. An acute exposure to Freon-22 can lead to neurological (headache, dizziness, and coma) and respiratory symptoms (sore throat, pharyngitis, and shortness of breath) [5]. In rare cases deaths have been associated with exposure to Freon [6, 7]. 

As mentioned above, severe vitamin B12 deficiency can manifest as different presentations such as anemia, neuropathy, optic atrophy, dementia, and myelopathy [1]. Amongst inhalant gases, nitrous oxide exposure has been known to cause cobalamin deficiency [8]. Nitrous oxide binds to cobalt component of coenzyme and converts the reduced form to oxidized form thus inactivating it [9]. In our case, patients clinical symptoms, which are typically seen in patients with B12 deficiency, and the findings on brain MRI have also been seen in patients with low B12 levels. In addition patient's symptoms resolved whenthe patient's B12 levels became normal. The intervention of limiting exposure to Freon gases via the use of mask resulted in normalization of patient's B12 levels and subsequently the levels dropped again with removal of the mask. 

Based on these findings and our extensive search in Pubmed and Medline databases, this is the first reported case of vitamin B12 deficiency with typical clinical symptoms in the setting of expsoure to CFC and other Freon gases. The exact mechanism, how this exposure results in B12 deficiency, is unclear but possibly may share some of the same common pathway as suggested with exposure to nitrous oxide. We feel that no inferences can be drawn from a single case report but our case suggests that CFC and other Freon gases and B12 levels may be closely linked and merit further research.
